# Incorporation of podoplanin into HIV released from HEK-293T cells, but not PBMC, is required for efficient binding to the attachment factor CLEC-2

**DOI:** 10.1186/1742-4690-7-47

**Published:** 2010-05-19

**Authors:** Chawaree Chaipan, Imke Steffen, Theodros Solomon Tsegaye, Stephanie Bertram, Ilona Glowacka, Yukinari Kato, Jan Schmökel, Jan Münch, Graham Simmons, Rita Gerardy-Schahn, Stefan Pöhlmann

**Affiliations:** 1 Nikolaus-Fiebiger-Center for Molecular Medicine, University Hospital Erlangen, 91054 Erlangen, Germany; 2 Institute for Clinical and Molecular Virology, University Hospital Erlangen, 91054 Erlangen, Germany; 3 Institute of Virology, Hannover Medical School, 30625 Hannover, Germany; 4 The Global COE Program for Medical Sciences, Japan Society for the Promotion of Science, The Oncology Research Center, Research Institute for Advanced Molecular Epidemiology, Yamagata University, 2-2-2 Iida-nishi, Yamagata 990-9585, Japan; 5 Institute of Molecular Virology, University Hospital Ulm, 89081 Ulm, Germany; 6 Blood Systems Research Institute and Department of Laboratory Medicine, University of California, San Francisco, CA, USA; 7 Cellular Chemistry, Center for Biochemistry, Hannover Medical School, 30625 Hannover, Germany

## Abstract

**Background:**

Platelets are associated with HIV in the blood of infected individuals and might modulate viral dissemination, particularly if the virus is directly transmitted into the bloodstream. The C-type lectin DC-SIGN and the novel HIV attachment factor CLEC-2 are expressed by platelets and facilitate HIV transmission from platelets to T-cells. Here, we studied the molecular mechanisms behind CLEC-2-mediated HIV-1 transmission.

**Results:**

Binding studies with soluble proteins indicated that CLEC-2, in contrast to DC-SIGN, does not recognize the viral envelope protein, but a cellular factor expressed on kidney-derived 293T cells. Subsequent analyses revealed that the cellular mucin-like membranous glycoprotein podoplanin, a CLEC-2 ligand, was expressed on 293T cells and incorporated into virions released from these cells. Knock-down of podoplanin in 293T cells by shRNA showed that virion incorporation of podoplanin was required for efficient CLEC-2-dependent HIV-1 interactions with cell lines and platelets. Flow cytometry revealed no evidence for podoplanin expression on viable T-cells and peripheral blood mononuclear cells (PBMC). Podoplanin was also not detected on HIV-1 infected T-cells. However, apoptotic bystander cells in HIV-1 infected cultures reacted with anti-podoplanin antibodies, and similar results were obtained upon induction of apoptosis in a cell line and in PBMCs suggesting an unexpected link between apoptosis and podoplanin expression. Despite the absence of detectable podoplanin expression, HIV-1 produced in PBMC was transmitted to T-cells in a CLEC-2-dependent manner, indicating that T-cells might express an as yet unidentified CLEC-2 ligand.

**Conclusions:**

Virion incorporation of podoplanin mediates CLEC-2 interactions of HIV-1 derived from 293T cells, while incorporation of a different cellular factor seems to be responsible for CLEC-2-dependent capture of PBMC-derived viruses. Furthermore, evidence was obtained that podoplanin expression is connected to apoptosis, a finding that deserves further investigation.

## Background

The envelope protein (Env) of the human immunodeficiency virus (HIV), a heavily glycosylated type I transmembrane protein, mediates infectious viral entry into target cells [1]. This process depends on the interactions of Env with proteins displayed at the surface of host cells. All primary HIV-1 isolates characterized to date engage the CD4 protein as receptor for infectious entry [2,3]. Upon binding to CD4, a coreceptor binding site is generated or exposed in Env, which allows engagement of the chemokine coreceptors CCR5 and CXCR4. The interactions of Env with CD4 and coreceptor are essential for infectious entry, and the interacting surfaces are key targets for preventive and therapeutic approaches [2,3]. For instance, a small molecule inhibitor of Env binding to CCR5, maraviroc, blocks spread of CCR5-tropic HIV and is used as salvage therapy for patients who do not respond to conventional HIV therapy [4,5].

Receptor expression levels can limit HIV entry into host cells [6,7], and this limitation can be overcome by concentrating virions onto target cells by, for example, centrifugation or polybrene treatment [8]. A constantly accumulating body of evidence suggests that certain host cell factors can also promote viral attachment to cells and can thereby increase infection efficiency [9,10]. A striking example is the interaction of HIV with a semen-derived fragment of prostatic acidic phosphatase, termed SEVI (for Semen Enhancer of Virus Infection) [11]. SEVI, an amyloidogenic peptide, forms fibrils in human semen which capture HIV and concentrate virions onto target cells [11]. As a consequence, SEVI boosts viral infectivity and might increase the risk of acquiring HIV infection upon sexual intercourse. Incorporation of host cell factors into the HIV envelope can also increase viral infectivity. The augmentation of infectivity is due to the interaction of the virion-incorporated factors with their cognate receptors on HIV target cells, as exemplified by the up to 100-fold increased infectivity of ICAM-1-bearing viruses for LFA-1 positive target cells [12,13]. Finally, attachment of HIV to dendritic cells can also promote HIV infection of adjacent T-cells [14,15], and this property has been associated with the expression of DC-SIGN [16], a calcium-dependent (C-type) lectin which recognizes mannose-rich carbohydrates on the HIV Env protein [17-19]. Engineered expression of DC-SIGN on certain cell lines promotes receptor-dependent infection of these cells (termed infection in cis) [20] or of adjacent target cells (termed infection in trans, or transmission) [16], and it has been suggested that DC-SIGN might promote HIV spread in and between individuals [16]. However, this hypothesis is intensely debated [21-25]. In fact, several lines of evidence suggest that DC-SIGN might mainly function as a pathogen recognition receptor, which promotes HIV uptake for MHC presentation and thereby exerts a protective function against HIV infection [23-27].

We and others have previously shown that apart from dendritic cells, platelets also express DC-SIGN and that these cell fragments bind to HIV in a mainly DC-SIGN-dependent manner [28,29]. However, the HIV binding activity of platelets could be partially inhibited by antisera specific for the newly identified HIV attachment factor CLEC-2 [29], indicating that CLEC-2 contributes to HIV capture by platelets. CLEC-2 is a lectin-like protein, and its putative carbohydrate recognition sequence contains 17 amino acid residues highly conserved between C-type lectins [30]. Binding of the snake venom toxin rhodocytin to CLEC-2 triggers Syk-dependent signalling in platelets which causes platelet degranulation [31,32]. Residues in CLEC-2 which are required for binding to rhodocytin have been defined [33,34]. However, it is at present unclear how CLEC-2 interacts with HIV.

Here, we report that CLEC-2, unlike DC-SIGN, does not bind to the viral Env protein, but to a cellular factor incorporated into the viral envelope. For viruses produced in the kidney-derived cell line 293T, this factor was found to be podoplanin (also termed aggrus), a cellular mucin-like glycoprotein expressed by kidney podocytes (which are known to be susceptible to HIV infection [35]) and lymphatic endothelium [36-38]. Podoplanin expression was not detected on viable, but on apoptotic T-cells and on apoptotic peripheral blood mononuclear cells (PBMCs). However, apoptosis of HIV infected T-cells was not associated with podoplanin expression. Nevertheless, CLEC-2 mediated trans-infection of HIV generated in PBMCs, indicating that these cells might express a so far unidentified CLEC-2-ligand which can facilitate CLEC-2-dependent HIV capture.

## Methods

### Cell culture and transfection

293T, 293 T-REx [19], GP2 293 (Clontech, California, USA) and CHO cells were maintained in Dulbecco's modified Eagle medium (DMEM) supplemented with 10% fetal calf serum (FCS, Biochrom, Germany), penicillin and streptomycin. In addition, blasticidin and zeocin were used for selection of 293 T-REx cells expressing CLEC-2 upon induction with doxycycline (Sigma, Germany). CHO Lec1 and CHO Lec2 cells [39-41] were cultured in αMEM (PAA, Germany), supplemented with 10% FCS and antibiotics. B-THP, B-THP DC-SIGN, B-THP CLEC-2 (Raji B cells that were engineered to express DC-SIGN [42], CLEC-2 [29] or empty vector), C8166-SEAP cells [43] and CEM×174 5.25 M7 (abbreviated CEM×174 R5) cells [44], the latter expressing exogenous CCR5, were cultured in RPMI 1640 medium (PAA, Germany) in the presence of antibiotics and 10% FCS. All cells were cultured at 37°C and 5% CO_2_. Highly purified platelets were obtained from the "Transfusionsmedizinische und Hämostaseologische Abteilung" of the University Hospital Erlangen. Alternatively, platelets were prepared from whole blood by centrifugation at 1200 rpm at RT. The upper platelet-rich plasma was collected and centrifuged at 4000 rpm for 20 min at RT. Subsequently, the supernatant was removed, and platelets were resuspended in RPMI 1640 medium supplemented with 10% FCS and antibiotics. PBMCs were isolated from whole blood or leukocyte filters by centrifugation through a Ficoll gradient and either cultured in RMPI 1640 medium supplemented with 10% FCS and antibiotics or stimulated with PHA (Sigma) at a concentration of 5 μg/ml and IL-2 (Roche) at a concentration of 10 U/ml.

### Plasmids

The NL4-3-based reporter virus bearing EGFP in place of *nef *was generated by splice overlap extension (SOE) PCR. Briefly, a NL4-3 env fragment was amplified using oligonucleotides pJM206 (binding upstream of the singular HpaI restriction site in env), and pJM394 (binding to the 3' end of *env *and also containing the first three triplets of EGFP) and pBRNL4-3 [45] as template. EGFP was amplified from pEGFP-C1 (Clontech) using primers JM395 (binding to EGFP start sequences) and JM396 (introducing a MluI site downstream of the EGFP stop codon). Both PCR fragments were fused by SOE PCR using primers pJM206 and pJM396. The resulting env-EGFP fragment was cloned via HpaI and MluI into pBRNL4-3_nef+ Δ1Δ2 [46] resulting in the generation of pBRNL4-3-EGFP in which *nef *was replaced by EGFP. Oligonucleotide sequences (env sequences in bold; EGFP sequences in italics, MluI restriction site underlined): pJM206 5'-**TGGAACTTCTGGGACGCAGG**-3'; pJM394 5'-***GCTCACCAT CTTA*TAGCAAAATCC;**JM395 **GCTATA*A***G***ATGGTGAGCAAGGGCG***-3';JM396 5'-CG**TACGCGTTACTTGTACAGC**-3'. The gp120-Fc-IgG_1 _construct [47] was generated by amplifying a codon-optimized gp120 (JRFL) [48] with primers gp120_BamHI 5'-GAGT*GGATCC*CTTATCGTCGTCATCCTTGTAATCC-3' (sense) and gp120_HindIII 5'-GTACG*AAGCTT*GTGGAGAAGCTGTGGGTGAC-3' (antisense), followed by insertion of the PCR fragment in the BamHI and HindIII restriction sites of the Fc-IgG_1 _encoding plasmid pAB61 [49]. For generating the CLEC-2-Fc-IgG_1 _fusion construct, sense primer 5'-GTACG*AAGCTT*TGCAGCCCCTGTGACACAAAC-3'and antisense primer 5'-GAGT*GGATCC*AGGTAGTTTCCACCTTGG-3' were used for PCR amplification, and the product was cloned into pAB61 using the HindIII and BamHI restriction sites. CLEC-2 mutants bearing single amino acid changes were generated by overlap extension PCR. The oligonucleotides 5'**-**GCC*GGATCC*ACCATGCAGGATGAAGATGGATACATC-3' (sense) and GCC*GAATTC*TTAAGGTAGTTGGTCCACCTTGG (antisense) were used as outer primers and combined with the following inner primers:5'-GATGGAAAAGGAGCCATGAATTGTGC-3' (sense) and 5'-AGCACAATTCATGGCTCCTTTTCCAT-3' (antisense) for generation of mutant CLEC-2 N192A, 5'-TTGAGTTTTTGGCCGATGGAAAAGG-3' (sense) and 5'-TCCTTTTCCATCGGCCAAAAACTCA-3' (antisense) for mutant CLEC-2 E187A, 5'-GTTTTTGGAAGATGGAGCCGGAAATATGAATTGTG-3' (sense) and 5'-AATTCATATTTCCGGCTCCATCTTCCAAAA-3' (antisense) for mutant CLEC-2 K190A, 5'-GCAACATTGTGGAATATATTGCGGCGCGCACCCATCTGATTC-3' (sense) and 5'-GCGCCGCAATATATTCCACAATG-3' (antisense) for mutant CLEC-2 K150A. For generation of DC-SIGN-Fc-IgG_1_, primers 5'-GTACG*AAGCTT*GAACGCCTGTGCCACCCCTG-3' (sense) and 5'-GAGT*GGATCC*CGCAGGAGGGGGGTTTGGGG-3' (antisense) were used. The resulting PCR fragment was cloned into pAB61, using the HindIII and BamHI restriction sites. A PCR fragment encoding the extracellular domain of podoplanin fused to the Fc portion of human immunoglobulin was generated as described above, employing primers 5**'-**GCC*AAGCTT*GCCAGCACAGGCCAGCCAGAAGATG-3' (sense) and 5'**-**GCG*GGATCC*TGTTGACAAACCATCTTTCTCAAC-3' (antisense) and inserted into the pAB61 plasmid via the HindIII and BamHI restriction sites (italics). The identity of all PCR amplified sequences was confirmed by sequencing with an ABI3700 genetic analyzer (Applied Biosystems) according to the manufacturer's instructions. The plasmid used for transient expression of podoplanin (podoplanin in pcDNA3) has been previously described [38].

### Viruses and transmission analyses

Replication-competent HIV-1 NL4-3, NL4-3 luc [50] and NL4-3 EGFP were generated as described elsewhere [50]. Briefly, 293T cells were transfected with plasmids encoding proviral DNA, and culture medium was changed 12 h post transfection. Culture supernatants were harvested at 48 h post transfection and filtered through a 0.45 μm filter, aliquoted and stored at -80°C. Transmission analyses were carried out as described [29]. Briefly, B-THP control cells, B-THP-DC-SIGN and B-THP-CLEC-2 cells [29,42] or platelets were incubated with virus for 3 h at 37°C, and unbound virus was removed by washing with fresh culture medium. Cells were then incubated with CEM×174 R5 target cells and luciferase activities in cellular lysates were determined three days after the start of the cocultivation by employing a commercially available system (Promega, Germany).

### Binding studies with soluble proteins

For generating soluble Zaire Ebolavirus glycoprotein (ZEBOV-GP)-Fc- [51], DC-SIGN-Fc-, CLEC-2-Fc- and Podoplanin-Fc-fusion proteins, 293T cells were calcium phosphate-transfected with the respective plasmids or pAB61 control plasmid encoding only the Fc-portion of IgG1. For transfection of CHO and mutant cell lines, Lipofectamine 2000 transfection reagent (Invitrogen, Germany) was used according to the manufacturer's protocol. The cells were washed with PBS and the culture medium was replaced by FCS-free medium at 12 h post-transfection and supernatants were harvested 48 h post-transfection. Subsequently, supernatants were concentrated using Centricon Plus-20 size-exclusion centrifugal filters (Millipore, Germany; centrifugation at 4000 g for 15 minutes), aliquoted, and stored at -80°C. To employ comparable amounts of soluble proteins for binding studies, Fc-fusion protein preparations were normalized by Western blot, employing an anti-human IgG-horseradish peroxidase conjugate for detection (Dianova, Germany). To assess binding, 5 × 10^5 ^cells were incubated with Fc-fusion proteins and Fc-control protein at 4°C for 45 minutes. Subsequently, the cells were washed with FACS buffer and stained with Cy5-conjugated anti-human IgG secondary antibody for 30 minutes at 4°C. Cell-staining was then analyzed by flow cytometry, employing a Cytomics FC500 flow cytometer (Beckman-Coulter, Florida, USA), and data were analyzed with FCS Express FACS analysis software (De Novo Software, Los Angeles, USA).

### Analysis of podoplanin surface expression

Analyses of podoplanin surface expression were performed by flow cytometry, using the podoplanin specific antibodies NZ-1 or 18H5 (Acris, Germany) in combination with secondary anti rat/mouse antibody coupled to Cy5 (Dianova, Germany). Cells were incubated with 10 μg/ml antibody in PBS supplemented with 5% FCS for 30 minutes at 4°C. Subsequently, PBS supplemented with 5% FCS was added, and the cells were pelleted by centrifugation (1200 rpm, 4°C for 5 minutes). Finally, cells were resuspended in fixans (1.5% paraformaldehyde) and incubated for 30 minutes at 4°C before staining was analyzed by flow cytometry. For all measurements 20,000 gated events were collected.

### Knock-down of podoplanin expression by shRNA

For stable knock-down of podoplanin in 293T cells, shRNAs were constructed by using shRNA Hairpin Oligonucleotide Sequence Designer Tool (Clontech, California, USA). The podoplanin specific shRNA 137 contained the target shRNA sequence, a hairpin loop region "TTCAAGAGA" and an antisense shRNA sequence followed by a pol III terminator sequence. The shRNA was constructed by annealing shRNA137sense_BamHI: 5'*GATCC*GCGAAGATGATGTGGTGACTTTCAAGAGAAGTCACCACATCATCTTCGTTTTTTACGCGTG3' and shRNA137antisense_EcoRI: 5'*AATTC*ACGCGTAAAAAACGAAGATGATGTGGTGACTTCTCTTGAAAGTCACCACATCATCTTCGCG3' followed by insertion of the double stranded fragment into the retroviral vector pSIREN-IRES-EGFP-RetroQ [52], using restriction enzymes BamHI and EcoRI, respectively. This vector allows stable expression of small hairpin RNAs in transduced cells, which can be readily identified and selected due to vector encoded genes for puromycin resistance and EGFP (enhanced green fluorescence protein) expression. Retroviral transduction was performed by transient expression of the shRNA constructs and VSV-G in the packaging cell line GP2-293 (Clontech, California, USA). At 48 h post transfection, cell supernatants were harvested, and viruses were concentrated by ultracentrifugation for 2 h at 4°C. Pelleted virions were resuspended in 2 ml medium containing 2 μg/ml polybrene (Sigma-Aldrich, Germany) and were used for transduction of 1 × 10^6 ^293T cells. At 24 h post transduction, cells were washed and incubated for 3 days. Subsequently, transduced cells were selected in medium containing 10 μg/ml puromycin (Sigma-Aldrich, Germany).

### Apoptosis induction

For apoptosis induction cells were incubated with 1 μM staurosporine (New England Biolabs, Germany), 25 μg/ml cycloheximide (Sigma-Aldrich, Germany) or 0.1% DMSO as a control in culture medium for 14 h unless otherwise stated. Cells were stained for apoptosis with PE-conjugated annexin V (R&D Systems, Minnesota, USA) and for necrosis with 7-aminoactinomycin D (7-AAD, Sigma, Germany). Specifically, cells were incubated with 5 μl annexin V or 7-AAD for 20 min at room temperature and then washed with PBS supplemented with 5% FCS. Subsequently, cells were fixed in 1.5% paraformaldehyde for 30 minutes at 4°C. Staining was analyzed within 30 minutes after completion of fixation by flow cytometry. For all measurements 20,000 gated events were collected.

### Inhibition of antibody binding by soluble podoplanin

The podoplanin specific antibodies 18H5 and NZ-1 (Acris, Germany) were pre-incubated with concentrated, soluble podoplanin-Fc fusion protein for 30 minutes at 4°C before staining of apoptotic cells for subsequent FACS analysis.

### Statistical analyses

Statistical significance was determined by employing a two-tailed student's t-test for paired samples.

## Results

### Efficient binding of soluble CLEC-2 to 293T cells does not require expression of the HIV-1 envelope protein

**Figure 1:**
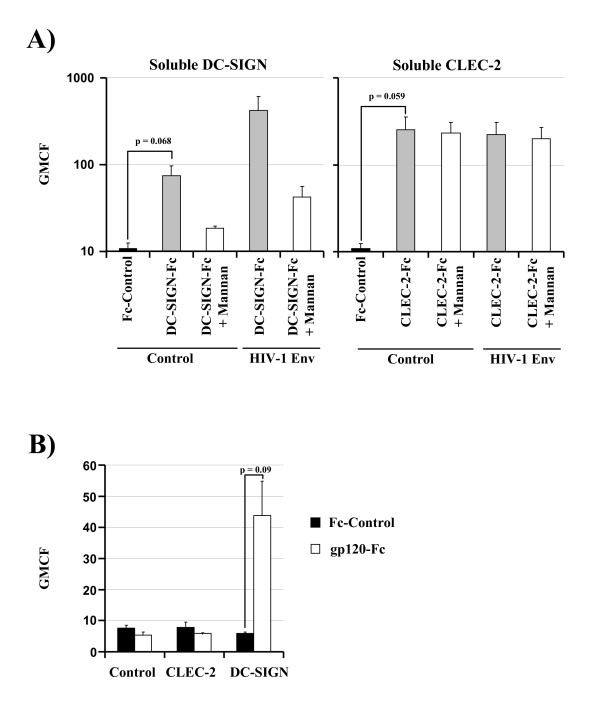
**CLEC-2 does not recognize the viral Env protein**. (A) 293T cells were either control transfected with empty vector or transfected with an HIV-1 NL4-3 Env expression plasmid. Subsequently, the cells were preincubated with PBS or mannan and then DC-SIGN-Fc (left panel) or CLEC-2-Fc (right panel) fusion proteins or an Fc-control protein (black bars) were added. Unbound proteins were removed by washing and bound proteins detected by flow cytometry. The results represent the average of the geometric mean channel fluorescence (GMCF) measured in four independent experiments. Error bars indicate standard error of the mean (SEM). (B) 293T cells were transfected with DC-SIGN, CLEC-2 or empty vector and incubated with soluble HIV-1 Env gp120-Fc fusion protein or control Fc-protein. Unbound proteins were removed by washing and bound proteins detected by flow cytometry. The results represent the average ± SEM of the GMCF measured in three independent experiments. GMCF: geometric mean channel fluorescence, SEM: standard error of the mean.

In order to better understand HIV-1 interactions with CLEC-2, we first asked if CLEC-2, like DC-SIGN [16], binds to the HIV-1 envelope protein (Env). For this, we generated soluble versions of DC-SIGN and CLEC-2 by fusing the extracellular domain of these lectins to the Fc-portion of human immunoglobulin. Soluble DC-SIGN bound to control transfected 293T cells with higher efficiency than the Fc-control protein (Fig. 1A), most likely due to recognition of cellular proteins harbouring high-mannose and/or fucose containing glycans, which are bound by DC-SIGN [17-19]. Notably, however, binding was substantially enhanced upon expression of the HIV-1 NL4-3 Env protein on 293T cells (Fig. 1A), indicating that DC-SIGN binds to HIV-1 Env, as expected from published data [16]. Finally, the interaction of soluble DC-SIGN with control cells and Env expressing cells was specific, since binding could be inhibited by the mannose-polymer mannan, a previously described inhibitor of DC-SIGN interactions with ligands [16]. Soluble CLEC-2 also bound to 293T cells with higher efficiency than the Fc-control protein (Fig. 1A). However, in stark contrast to the results obtained with soluble DC-SIGN, the interaction was not inhibited by mannan and was not enhanced by expression of the viral Env protein. In agreement with these results, soluble HIV-1 Env protein bound specifically to DC-SIGN but not to CLEC-2 expressing cells (Fig. 1B). We therefore concluded that CLEC-2, in contrast to DC-SIGN, does not capture HIV-1 Env. Instead, CLEC-2 seemed to recognize a cellular factor expressed on 293T cells, and binding to this factor did not depend on recognition of high-mannose carbohydrates.

### Podoplanin, a recently identified CLEC-2 ligand, is expressed on 293T cells

**Figure 2:**
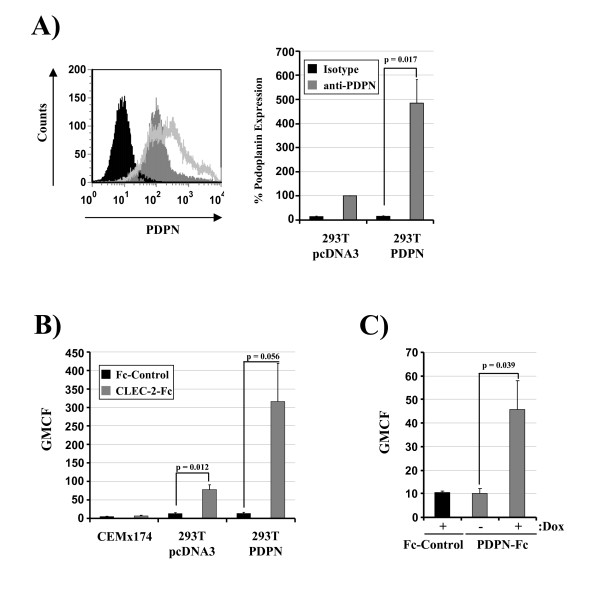
**Podoplanin is expressed on 293T cells and binds to CLEC-2**. (A) 293T cells were either control transfected with empty vector or transfected with a podoplanin expression construct. Cells were stained with anti-podoplanin antibody 18H5 and analyzed by flow cytometry (black filled area: control transfected cells stained with isotype antibody, grey filled area: control transfected cells stained with 18H5, grey line: cells transfected with podoplanin expression plasmid and stained with 18H5). The results of a representative experiment are shown on the left side, the average of four independent experiments is presented at the right side. Error bars indicate SEM. (B) The experiment was carried out as described for (A), but binding of soluble CLEC-2 to podoplanin or control transfected cells and to CEM×174 cells was analyzed. The results represent the average ± SEM of the GMCF measured in three (CEM×174) and four (293T, 293T-PDPN) independent experiments. (C) 293 T-REx CLEC-2 cells were doxycycline treated to induce CLEC-2 expression or PBS treated, and binding of soluble podoplanin-Fc or Fc-control protein was analyzed. The results represent the average ± SEM of the GMCF measured in three independent experiments. Dox: doxycycline, GMCF: geometric mean channel fluorescence, PDPN: podoplanin, SEM: standard error of the mean.

The cellular mucin podoplanin was recently shown to interact with CLEC-2 [53]. Podoplanin is endogenously expressed by kidney podocytes [37]. Therefore, we investigated if the kidney-derived cell line 293T also expresses podoplanin. Flow cytometric analysis indeed revealed high levels of podoplanin on the surface of 293T cells (Fig. 2A). Expression was further enhanced upon transfection of 293T cells with a podoplanin expression plasmid (Fig. 2A), and higher levels of podoplanin resulted in more efficient binding of soluble CLEC-2 (Fig. 2B). In contrast, no binding to the lymphoid cell line CEM×175 R5 was detected (Fig. 2B), which was podoplanin negative (see below). We then used soluble podoplanin to confirm the interaction with CLEC-2. For this, CLEC-2 expression was induced on 293 T-REx CLEC-2 cells, and binding of soluble podoplanin fused to the Fc-portion of human immunoglobulin was analyzed by flow cytometry. Efficient binding of soluble podoplanin was observed only upon induced expression of CLEC-2, and a control Fc protein did not bind to the CLEC-2 expressing cells (Fig. 2C and data not shown). Thus, 293T cells, which we and many others frequently use for production of HIV-1 stocks, express podoplanin; and podoplanin specifically interacts with CLEC-2.

### Glycosylation of podoplanin is required for efficient binding to CLEC-2

**Figure 3:**
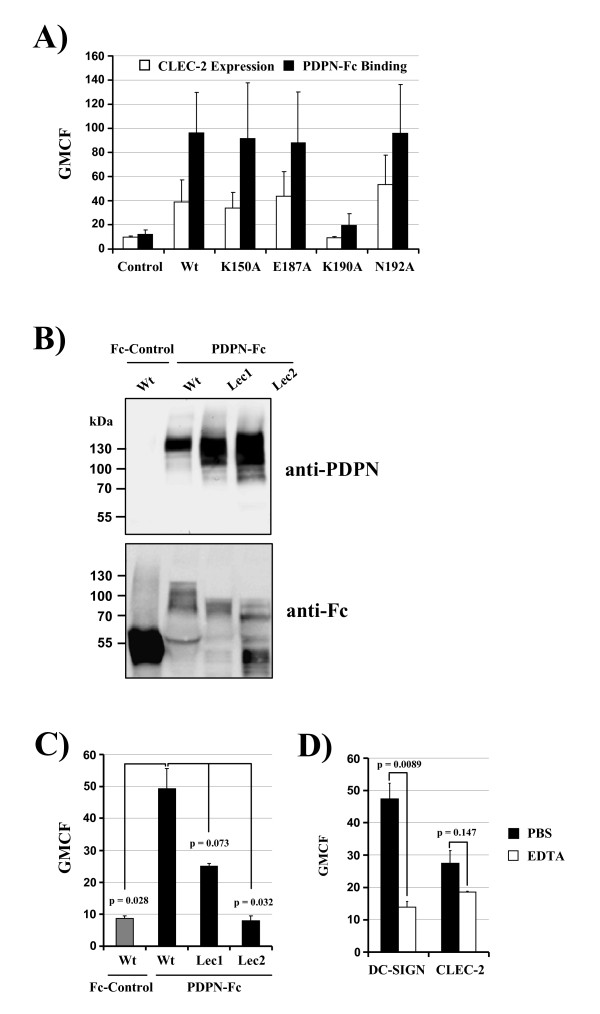
**Binding of podoplanin to CLEC-2 requires adequate podoplanin glycosylation and is independent of divalent ions**. (A) The indicated CLEC-2 mutants were transiently expressed on 293T cells and expression (white bars) and binding of podoplanin-Fc (black bars) analyzed by flow cytometry. The results represent the average ± SEM of the GMCF measured in three independent experiments. (B) The Fc-control protein or the podoplanin-Fc fusion protein was transiently expressed in the indicated CHO cell lines. CHO Lec1 cells are defective in N-acetylglucosaminyltransferase (no complex N-glycans are generated), CHO Lec2 cells lack the CMP-sialic acid transporter (no sialylated glycoconjugates are generated). The supernatants of the transfected cells were harvested, concentrated and analyzed by Western blot, using the podoplanin-specific D2-40 antibody [82] (top panel) or a Fc-specific antibody (bottom panel). (C) The proteins generated in (B, control Fc-protein was 2-fold diluted) were incubated with CLEC-2 expressing 293 T-REx cells and bound protein was detected by FACS. The results represent the average ± SEM of the GMCF measured in three independent experiments. (D) Expression of DC-SIGN and CLEC-2 was induced on 293 T-REx cells by doxycycline treatment and the cells incubated with ZEBOV-GP-Fc or podoplanin-Fc, respectively, in the presence of PBS (dark bars) or 2 mM EDTA containing FACS buffer (white bars). Bound proteins were detected by flow cytometry. The results represent the average ± SEM of the GMCF measured in three independent experiments. GMCF: geometric mean channel fluorescence, PDPN: podoplanin, SEM: standard deviation of the mean.

We next sought to elucidate the determinants governing efficient interactions between podoplanin and CLEC-2. For instance, it is at present unclear if glycosylation of podoplanin is required for binding to CLEC-2. Watson and colleagues demonstrated that binding of CLEC-2 to the snake venom protein rhodocytin is glycosylation independent, and defined several amino acids in CLEC-2 which contributed to efficient rhodocytin binding [33,34]. Thus, mutations K150A, E187A, K190A and N192A decreased binding of CLEC-2 to rhodocytin in surface plasmon resonance binding studies [34]. We addressed if these residues were also required for binding to soluble podoplanin. Flow cytometric analysis showed that all changes, with the exception of K190A were compatible with efficient expression of CLEC-2 (Fig. 3A). Wild type CLEC-2 and all mutants, except K190A, bound to soluble podoplanin with similar efficiency, indicating that the CLEC-2 residues involved in rhodocytin binding were not important for binding to podoplanin. Podoplanin contains sialylated *O*-glycans [54], and we next analyzed if glycosylation of podoplanin is essential for binding to CLEC-2. For this, podoplanin-Fc fusion proteins were produced in wt CHO cells or CHO cells that due to defects in either the medial Golgi localized *N*-acetylglucosaminyltransferase I (CHO Lec1) or the trans Golgi localized CMP-sialic acid transporter (CHO Lec2) have lost their abilities to produce complex *N*-glycans and sialylated glycoconjugates, respectively [39-41]. Soluble proteins were concentrated from cellular supernatants by size-exclusion filtration, and Western blot analysis showed that the podoplanin-Fc preparations contained roughly comparable amounts of protein (Fig. 3B), while the Fc-control protein preparation was more concentrated. When binding to CLEC-2 was analyzed in a FACS-based assay, podoplanin produced in Lec1 cells still bound to CLEC-2 with appreciable efficiency (Fig. 3C). In contrast, podoplanin produced in Lec2 cells and thus almost completely lacking sialoglycoconjugates did not show significant binding to CLEC-2 (Fig. 3C). The observed differences indicate that the presence of sialic acid is essential for binding to CLEC-2. Moreover, because *N*-glycans are exclusively of the high-mannose type if proteins are expressed in Lec1 cells, this finding provides evidence that sialylated *O*-glycans are involved in mediating the contact to CLEC-2. Based on the knowledge that EDTA influences binding properties of DC-SIGN [16], we next asked if also the interaction between CLEC-2 and podoplanin depends on divalent ions. As shown in Fig. 3D, treatment of DC-SIGN expressing cells with EDTA significantly reduced binding to soluble ZEBOV-GP-Fc, but had no effect on binding of soluble podoplanin to CLEC-2 (Fig. 3D), indicating that divalent ions are not required for the structural integrity of the podoplanin binding surface of CLEC-2.

### Podoplanin is incorporated into virions produced in 293T cells and virion incorporation is essential for CLEC-2-dependent HIV-1 interactions with cell lines and platelets

**Figure 4:**
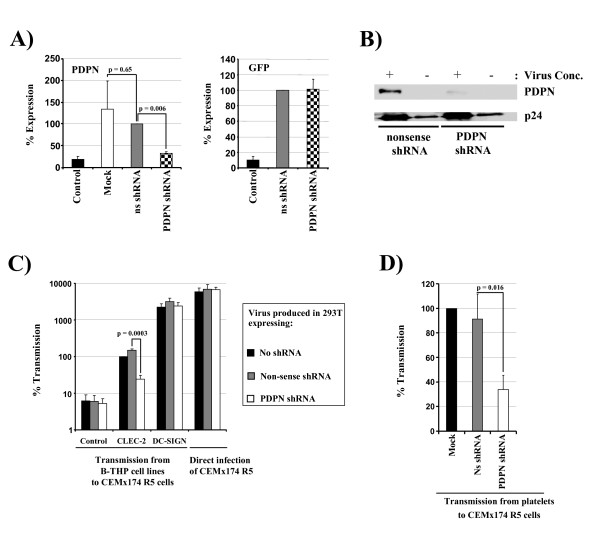
**Podoplanin is incorporated into virions released from 293T cells, and incorporation is essential for efficient CLEC-2-dependent HIV transmission**. (A) 293T cells were transduced with retroviral vectors encoding EGFP and either a podoplanin-specific or a non-sense shRNA. Transduced cells were puromycin-selected and podoplanin (left panel) and EGFP expression (right panel) was determined by flow cytometry (using antibody 18H5). The average ± SEM of five independent experiments, for which GMCF was determined, is presented. Podoplanin expression on cells expressing control shRNA was set as 100%. (B) An env-defective NL4-3 proviral genome was transiently expressed in 293T cells transduced with vector encoding either podoplanin-specific shRNA or non-sense shRNA; the supernatants were harvested, and either processed directly or concentrated by size-exclusion filtration. Subsequently, the supernatants were analyzed for podoplanin and p24-content by Western blot. (C) The cells described in (A) were transfected with HIV-1 NL4-3 proviral DNA; the supernatants were harvested and their p24-content determined. Equal volumes of virus stocks containing 10 ng of p24-antigen were then incubated with the indicated B-THP cell lines and bound viruses transmitted to CEM×174 R5 targets. In parallel, direct infection of targets was assessed. The results represent the average ± SEM of six independent experiments carried out in triplicates with two independent virus stocks. Transmission of HIV-1 produced in 293T cells not transduced with shRNA-encoding vector was set as 100%. Control indicates B-THP cells stably transduced with empty vector. (D) The experiment was conducted as described in (C). However, HIV-1 transmission by platelets was examined. The results represent the average ± SEM of five independent experiments carried out in triplicates. The same virus stocks as in (C) were used. Mock indicates viruses produced in 293T cells not transduced with shRNA-encoding vector. GMCF: geometric mean channel fluorescence, ns shRNA: none-sense shRNA, PDPN: podoplanin, SEM: standard error of the mean.

Our results so far indicated that podoplanin is expressed by 293T cells and that podoplanin specifically interacts with CLEC-2. We next assessed if podoplanin is incorporated into HIV-1 released from transfected 293T cells and if the virion incorporation of podoplanin is required for HIV-1 interactions with CLEC-2. To address these questions, particularly the potential relevance of podoplanin for HIV-1 interactions with CLEC-2, we employed shRNA knock-down. We first tested a panel of podoplanin-specific shRNAs and identified one shRNA which efficiently reduced podoplanin expression in transiently transfected 293T cells (data not shown). Subsequently, this shRNA was stably introduced into 293T cells by employing a retroviral vector, which also contained an expression cassette for EGFP. As control, cells were transduced with a retroviral vector encoding a non-sense shRNA. After cultivation in selection antibiotics, all cells were positive for EGFP and thus harboured the vector genome (Fig. 4A). Podoplanin expression was not appreciably altered in cells containing the vector encoding the control shRNA. In contrast, cells transduced with the vector encoding the podoplanin-specific shRNA showed substantially (~70%) reduced podoplanin expression (Fig. 4A), indicating that the shRNA was active. Next, we tested if podoplanin was incorporated into virions released from control cells and from the podoplanin knock-down cells. For this, the cells were transfected with env-deficient HIV-1 proviral DNA (for augmented biosafety), the supernatants concentrated by size-exclusion filtration and virions pelleted by centrifugation through a sucrose cushion. Alternatively, unconcentrated supernatants were directly passed through a sucrose cushion. Western blot analysis of these virion preparations yielded a prominent podoplanin signal for virions generated in control cells and a faint signal for virions generated in podoplanin knock-down cells (Fig. 4B). These signals were only observed for concentrated virions, and assessment of p24 content showed that concentration of particles was indeed effective (Fig. 4B). Finally, a markedly higher podoplanin signal was measured in the supernatants of HIV transfected compared to mock transfected cells (data not shown), confirming that the podoplanin signal observed in Fig. 4B was mainly due to virion-associated protein. Thus, podoplanin is incorporated into particles generated from 293T cells and incorporation can be reduced by shRNA-mediated knock-down. We then asked if reduced podoplanin incorporation affects HIV-1 interactions with CLEC-2. For this, virions were generated in control and podoplanin knock-down cells, normalized for p24-content and analyzed in trans-infection experiments. Reduction of virion-incorporation of podoplanin had no effect on DC-SIGN-dependent HIV-1 transmission by B-THP cells [42] (Fig. 4C), and infection experiments confirmed that the viruses employed were of comparable infectivity for target cells (Fig. 4C) and did not infect the transmitting cells (data not shown). In contrast, diminished podoplanin incorporation resulted in a pronounced reduction of viral transmission by CLEC-2 expressing B-THP cells and by platelets (Fig. 4C-D), demonstrating that podoplanin incorporation into virions produced in 293T cells is required for efficient interaction with CLEC-2.

### Reactivity of apoptotic cells with podoplanin-specific antibodies

**Figure 5:**
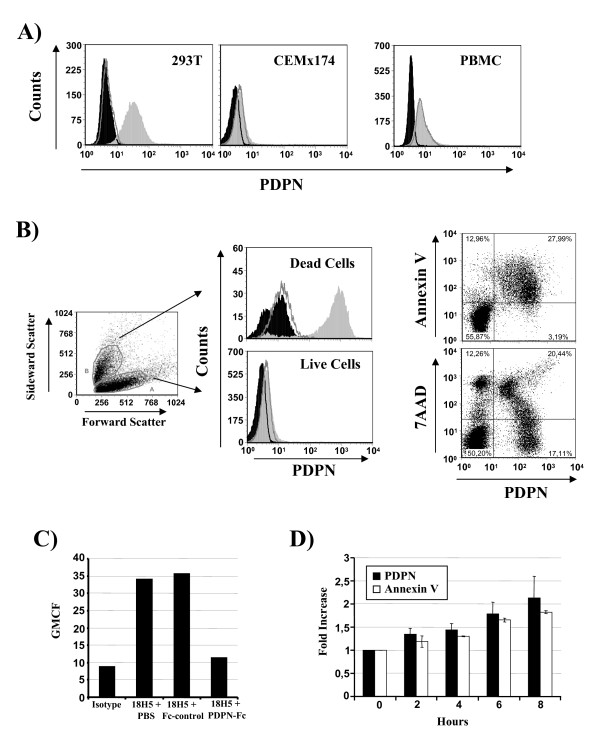
**Evidence that apoptotic cells express podoplanin**. (A) Podoplanin expression on the indicated cell lines was assessed by flow cytometry. Black filled histogram: unstained cells, grey line: cells stained with isotype control antibody, grey filled histogram: cells stained with 18H5. Similar results were obtained in two independent experiments. (B) Apoptotic and necrotic CEM×174 express podoplanin. Cultured CEM×174 cells were serum-starved and podoplanin expression on viable and apoptotic cells, as determined by forward and sideward scatter, analyzed by flow cytometry (left panel, the histograms were obtained by gating on dead or live cells, as indicated by the arrows in the scatter plot). Alternatively, the cells were co-stained with podoplanin-specific antibody and the apoptosis marker annexin V or the necrosis marker 7-AAD, and staining analyzed by flow cytometry including both, live and dead cells. Black filled histogram: unstained cells, grey line: cells stained with isotype control antibody, grey filled histogram: cells stained with 18H5. (C) The podoplanin-specific antibody 18H5 was pre-incubated with podoplanin-Fc fusion protein or Fc-control protein before addition to apoptotic cells, and antibody binding was subsequently analyzed by flow cytometry. The results of a representative experiment are shown. Similar results were obtained in three separate experiments. (D) Serum-starved CEM×174 cells were incubated with 1 μM staurosporine for the indicated times and then stained with anti-podoplanin antibody 18H5 and annexin V. Subsequently, podoplanin expression (black bars) and annexin V binding (white bars) were determined by flow cytometry. The results represent the average of two independent experiments and are shown relative to the podoplanin expression and annexin V binding at 0 hrs. The error bars indicate SEM. GMCF: geometric mean channel fluorescence, SEM: standard error of the mean.

Podocytes, which are visceral epithelial cells of the kidney, express podoplanin and were found to be infected in HIV-1 patients and to proliferate in HIV-1 associated nephropathy [35]. We analyzed if major HIV-1 target cells also express podoplanin. Analysis of PHA/IL-2 stimulated PBMCs and the T/B-cell hybrid cell line CEM×174, which is permissive to HIV and SIV infection [55,56], yielded no evidence for podoplanin expression when cells were gated for viability (Fig. 5A). Unexpectedly, however, CEM×174 cells and PBMCs defined as non-viable by our gating strategy efficiently bound the podoplanin antibody 18H5 but not an isotype-matched control antibody (Fig. 5B and Additional file 1); note that CEM×174 cells were serum starved to increase the percentage of non-viable cells. Co-staining of CEM×174 cells with the apoptosis marker annexin V and the necrosis marker 7-aminoactinomycin D (7-AAD) revealed that virtually all apoptotic cells and roughly half of the necrotic cells reacted with the podoplanin antibody (Fig. 5B). Comparable results were obtained with PBMCs (see Additional file 1), albeit only a portion of the apoptotic cells also expressed podoplanin. Apoptosis can result in surface expression of proteins which are not found on the surface of viable cells [57,58]. It is thus possible that podoplanin expression is up-regulated during apoptosis. However, apoptosis can also non-specifically change antibody reactivity of cells [59]. To discern between these possibilities, we first asked if staining of non-viable cells was a specific feature of the particular antibody used for detection of podoplanin (clone 18H5). Notably, staining of apoptotic cells was also observed with a different podoplanin antibody (clone NZ-1 [60], data not shown), which was generated in a different species (rat) and binds to an epitope distinct from but overlapping with the one recognized by 18H5 [61]. In contrast, staining of apoptotic cells was not observed with several unrelated antibodies (see Additional file 2). Moreover, binding of both antibodies, 18H5 and NZ-1, to apoptotic cells could be inhibited by the pre-incubation of antibodies with soluble podoplanin before staining of cells whereas pre-incubation with a control protein had no effect on antibody binding (Fig. 5C), indicating that antibody reactivity was dependent on the availability of the antigen binding site. So far, we had only analyzed cells naturally undergoing apoptosis in culture. Therefore, we next asked if reactivity against podoplanin antibodies could be induced by triggering of apoptosis with staurosporine, a relatively non-selective protein kinase inhibitor isolated from Streptomyces staurospores [62]. Indeed, treatment of CEM×174 cells and PBMCs with staurosporine induced binding of annexin V and anti-podoplanin-specific antibodies 18H5 and NZ-1 (Fig. 5D and Additional file 1), underlining a potential link between apoptosis induction and podoplanin expression.

### Podoplanin is not expressed on HIV-1 infected T-cells

**Figure 6:**
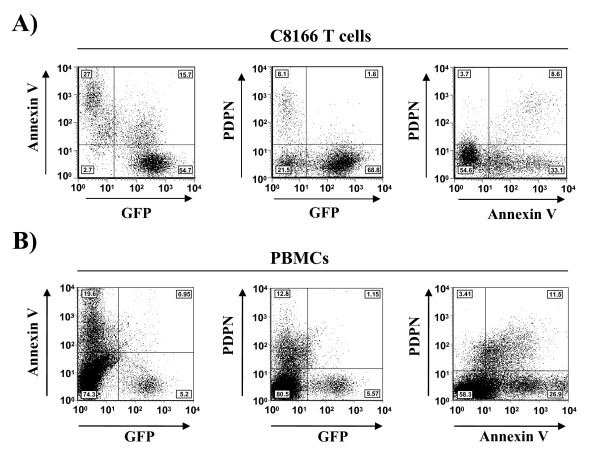
**Podoplanin is not expressed on HIV-infected T-cells**. (A) C8166-SEAP cells were infected with an HIV-1 NL4-3 variant bearing the EGFP gene in place of nef. At 7 days post infection the infected cells were stained with podoplanin-specific antibody 18H5 and annexin V and analyzed by flow cytometry. Similar results were obtained in an independent experiment. (B) The experiment was conducted as described in (A). However, PHA stimulated PBMCs were infected and stained. The results were confirmed in two separate experiments.

Apoptosis of infected and bystander cells is a prominent feature of HIV infection [63]. We therefore asked if podoplanin can be detected on HIV-1 infected C8166 T-cells and PBMCs or on uninfected bystander cells. For this, C8166-SEAP cells (Fig. 6A) and PBMCs (Fig. 6B) were infected with a replication-competent HIV-1 variant harbouring EGFP and analyzed for binding of annexin V and the podoplanin-specific antibody 18H5 at seven days post infection, when massive cytopathic effect was visible in infected C8166-SEAP cell cultures. Most HIV-1 infected cells did not react with annexin V (Fig. 6, left panel), in agreement with the published observation that HIV-1 infected cells maintain phospholipid asymmetry [64]. Likewise, infected cells did not bind the podoplanin-specific antibody (Fig. 6, middle panel). In contrast, podoplanin was readily detected on annexin V-positive cells (Fig. 6 right panel), which mainly represent uninfected bystander cells (Fig. 6, left panel). These observations suggest that podoplanin is not expressed on HIV-1 infected primary and immortalized T-cells and might thus play a limited role in cellular attachment of HIV-1 in infected patients.

### Viruses generated in PBMCs are transmitted by CLEC-2

**Figure 7:**
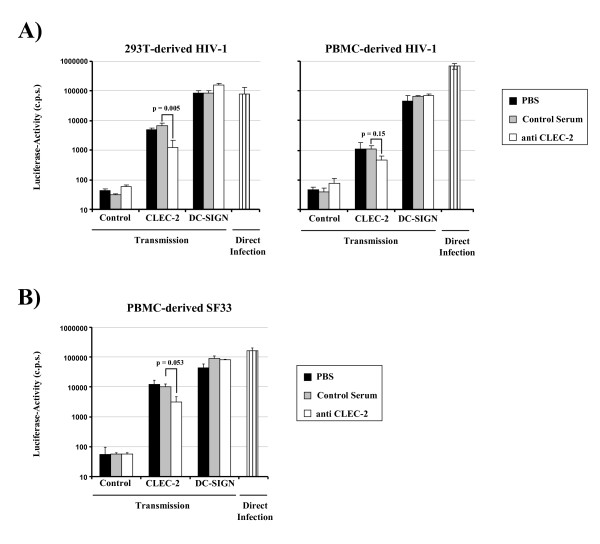
**HIV-1 produced in PBMCs is transmitted by CLEC-2**. (A) Stocks of HIV-1 NL4-3 were generated in 293T cells (left panel) and in PBMCs (right panel) and used for transmission and direct infection, employing the indicated cell lines, as described for figure 4. The results of representative experiments performed in triplicate are shown, error bars indicate SD. Similar results were obtained in two independent experiments (B) The experiment was carried out as described in (A) but transmission of HIV-1 SF33 generated in PBMC was analyzed. The results of a representative experiment performed in triplicate are presented and were confirmed in a separate experiment; error bars indicate SD. C.p.s.: counts per second, SD: standard deviation.

Our expression studies indicated that podoplanin is not expressed on stimulated, viable PBMCs and T-cell lines (Fig. 5), and that podoplanin expression is not induced in C8166 T-cells and PBMCs by HIV-1 infection (Fig. 5). These results raised the question if viruses generated in PBMCs are indeed transmitted in a CLEC-2-dependent fashion. Notably, B-THP CLEC-2 cells promoted trans-infection of HIV-1 NL4-3 (X4-tropic) produced in 293T cells and PBMCs, and these processes could be reduced by CLEC-2-specific antiserum (Fig. 7A). Likewise, HIV-1 SF33 (X4-tropic) generated in PBMCs was transmitted to T-cells by B-THP CLEC-2 cells, and transmission was inhibited by CLEC-2 specific antiserum to an extent which closely approached statistical significance (Fig. 7B), suggesting that viruses generated in PBMCs harbour a cellular factor which mediates binding to CLEC-2, but is different from podoplanin.

## Discussion

Several cellular lectins interact with the highly glycosylated HIV Env protein [16,25,65-67], and virus capture by these factors has been suggested to impact HIV spread in and between individuals [15,16,68]. We have previously reported that platelets, anucleated cell fragments which play an essential role in hemostasis, express the HIV attachment promoting proteins DC-SIGN and CLEC-2 [29]. Here, we show that DC-SIGN and CLEC-2 employ fundamentally different strategies to capture HIV. DC-SIGN binds to the HIV Env protein, while CLEC-2 recognizes (a) cellular factor(s) incorporated into HIV particles. The cellular mucin-like glycoprotein podoplanin was identified as such a factor, at least for virions generated in the widely used kidney-derived cell line 293T. Podoplanin was not expressed on viable T-cells, the major HIV target cell, and might thus be of minor importance for viral spread in vivo. Nevertheless, virions generated in PBMCs, which were found to be podoplanin negative, were transmitted to T-cells in a CLEC-2-dependent fashion, suggesting that PBMC-derived particles might harbour a so far undiscovered CLEC-2 ligand. Finally, a potential link between podoplanin expression and apoptosis was discovered which merits further investigation.

DC-SIGN recognizes mannose-rich carbohydrates on the surface of the HIV Env protein and requires Ca^++ ^ions for its structural integrity [16-19]. Consequently, DC-SIGN bound to soluble Env, binding of soluble DC-SIGN to 293T cells was strongly enhanced by expression of HIV Env, and ligand binding to DC-SIGN was prevented by the mannose-polymer mannan and chelators like EDTA (Fig. 1). In contrast, CLEC-2 did not recognize soluble HIV Env, binding of soluble CLEC-2 to 293T cells was not augmented by expression of HIV Env, and mannan and EDTA did not interfere with ligand binding to CLEC-2 (Fig. 1). These findings confirm our previous results obtained with virus-particles [29] and suggest that CLEC-2 does not recognize Env, but a host cell factor which is expressed on 293T cells. They also indicate that CLEC-2 is neither mannose-specific nor calcium-dependent. Thus, DC-SIGN and CLEC-2 differ profoundly in their mechanisms of ligand binding and in their ligand specificities.

The discovery of Suzuki-Inoue and colleagues [53] that podoplanin, a cellular mucin expressed on kidney podocytes [37], type I alveolar cells and lymphoid endothelial cells [36], binds to CLEC-2 and activates CLEC-2-dependent signalling, suggested that podoplanin might be the elusive CLEC-2 ligand on 293T cells. Indeed, FACS analysis revealed robust and homogenous podoplanin expression on 293T cells (Fig. 2), in agreement with recently published reports [69,70], and binding studies with soluble proteins confirmed that CLEC-2 and podoplanin interact (Fig. 2). Watson and colleagues previously defined amino acids in CLEC-2, which are important for the interaction with the snake venom component rhodocytin, and suggested that CLEC-2 binding to ligands might be carbohydrate-independent [33,34]. Notably, none of the amino acid residues important for rhodocytin binding was critical for efficient binding to podoplanin, while the presence of sialylated glycotopes on podoplanin was indispensable (Fig. 3), in agreement with previous results [54,71]. Rhodocytin and podoplanin might therefore engage CLEC-2 differentially, and a potential lectin-activity of CLEC-2 requires further investigation.

The endogenous expression of podoplanin on 293T cells and the specific interaction of podoplanin with CLEC-2 raised the questions if podoplanin was incorporated into virions produced in 293T cells, and if incorporation of podoplanin was required for CLEC-2 binding of these virions. Western blot analysis and knock-down of podoplanin expression by shRNA provided affirmative answers to both questions: Podoplanin depletion reduced CLEC-2-, but not DC-SIGN-, dependent HIV-1 transmission by B-THP cells, and diminished transmission by platelets by about 50% (Fig. 4). The latter finding is in agreement with our previous observation that CLEC-2-specific antiserum reduced HIV-1 transmission by platelets by about half [29]. Podoplanin therefore joins the list of host factors which can be incorporated into the HIV-1 envelope and impact HIV-1 infection by interacting with their cognate ligands [9,10]. A prominent example for such a factor is ICAM-1 which was found to be incorporated into the viral membrane, and to facilitate HIV-1 infection by binding to its ligand LFA-1 on T-cells [12].

The potential relevance of podoplanin incorporation for HIV spread in infected individuals is critically determined by the overlap of the podoplanin expression pattern with the cellular tropism of HIV. Analysis of T-cell lines and PBMCs for podoplanin expression yielded negative results (Fig. 5), at least when viable cells were analyzed (see below), indicating that HIV particles generated in patients might not harbour podoplanin. The exception might be viruses released from kidney podocytes which have been documented to express podoplanin [37] and to be susceptible to HIV infection [35]. However, the biological relevance of this process is questionable. In this context, it also needs to be noted that podoplanin expression is up-regulated in many tumours including Kaposi sarcoma [72,73]. Podoplanin/CLEC-2-dependent platelet stimulation by tumour cells promotes hematogenous tumour metastasis [71,74], possibly by inducing growth factor secretion by platelets and by promoting formation of a "platelet cap", which protects the tumour from mechanical forces. Thus, podoplanin might play a role in the development of the AIDS-associated Kaposi sarcoma, but is unlikely to modulate HIV spread in patients. Nevertheless, HIV-1 produced in PBMCs was transmitted to target cells in a CLEC-2-dependent fashion (Fig. 7), suggesting that primary T-cells might express a so far unrecognized CLEC-2 ligand (a hypothesis also raised by others [75]), which is incorporated into the viral envelope and which facilitates HIV transmission by CLEC-2. Our ongoing studies are devoted to the identification of this factor.

Podoplanin was not detected on viable CEM×174 cells (a T/B cell hybrid) and PBMCs, as determined by our gating strategy and by co-staining with the apoptosis and necrosis markers annexin V and 7-AAD, respectively (Fig. 5 and Additional file 1). In contrast, we observed efficient reactivity of two different podoplanin antibodies with non-viable cells, raising the intriguing possibility that podoplanin might be expressed at the cell surface in the context of apoptosis. Apoptosis can indeed alter expression of surface markers [57,58,76] but might also modulate antibody reactivity of cells [59], making the analyses of podoplanin expression by apoptotic cells a technically challenging task. Our findings that two antibodies, 18H5 and NZ-1, which were generated in different species and recognize different but overlapping epitopes in podoplanin [61], both specifically bind to apoptotic cells (Fig. 5 and data not shown), and that this reactivity depends on the availability of the antigen-binding site (Fig. 5C) suggests to us that binding is most likely specific. Furthermore, nested RT-PCR detected podoplanin message in CEM×174 cells (data not shown), suggesting low levels of podoplanin expression in these cells. Importantly, the podoplanin message did not appreciably increase upon apoptosis induction, and treatment with cycloheximide did not block specific staining of apoptotic cells with podoplanin antibodies (data not shown). Therefore, one must assume that podoplanin protein (or an antigenically related protein) is present within CEM×174 cells and other cell types, and that the protein becomes accessible to antibody staining only upon induction of apoptosis. If the latter process is due to specific transport of podoplanin to the cell surface or to membrane disintegration during apoptosis could not be conclusively determined. Regardless of the mechanism underlying reactivity of apoptotic cells with podoplanin-specific antibodies, podoplanin was not detected on HIV infected viable and apoptotic cells (Fig. 6), indicating that podoplanin expression is not altered in the context of HIV infection.

Collectively, our data help to understand how HIV interacts with CLEC-2, an HIV attachment factor on platelets. Several lines of evidence suggest that this interaction could impact HIV spread in infected patients. For one, thrombocytopenia (reduced platelet count) is frequent in HIV/AIDS patients [77], and it is conceivable that CLEC-2-dependent binding of HIV to platelets results in platelet clearance and thus contributes to reduced platelet counts. In addition, the interaction of HIV with CLEC-2 on platelets might induce platelet activation, which was found to be associated with HIV infection [78]. Moreover, CLEC-2-dependent HIV binding to platelets might result in trans-infection or virus degradation [28,29], and both processes could impact viral load and disease development. Finally, it is worth noting that liver sinusoidal endothelial cells and megakaryocytes also express CLEC-2 [66] and that both cell types are susceptible to HIV infection [79-81], which might be modulated by CLEC-2. In summary, CLEC-2 is expressed on several cell types exposed to HIV in patients and thus has the potential to modulate viral spread.

## Conclusions

Our results highlight that incorporation of cellular factors can alter HIV attachment to cells and cell to cell transmission. While podoplanin is unlikely to be incorporated into HIV particles produced in infected patients, our results indicate that HIV might incorporate a functional analogue of podoplanin in vivo, and that this process might promote virus binding to CLEC-2 positive cells. The identification of the respective factor and the clarification of the potential connection between podoplanin expression and apoptosis are interesting tasks for future research.

## Competing interests

The authors declare that they have no competing interests.

## Authors' contributions

CC analyzed binding of soluble CLEC-2 and soluble DC-SIGN to HIV Env, determined podoplanin expression by 293T cells, generated and characterized podoplanin knock-down cells, compared transmission of HIV generated in podoplanin-knock-down and control cells, and analyzed CLEC-2-dependent transmission of HIV generated in 293T cells and PBMCs, IS analyzed podoplanin expression on apoptotic cells, determined inhibition of antibody binding to apoptotic cells by soluble podoplanin, analyzed podoplanin expression on uninfected cell lines and HIV infected C8166-SEAP cells, TST analyzed binding of soluble podoplanin to CLEC-2 mutants, determined the impact of glycosylation and divalent ions on podoplanin binding to CLEC-2, analyzed podoplanin expression on cell lines and determined podoplanin incorporation into virions, SB and IG determined podoplanin RNA expression in apoptotic cells and analyzed podoplanin expression by viable cells, JS analyzed podoplanin expression on HIV infected PBMCs, YK provided critical reagents and contributed to the interpretation of experiments, JM, GS and RGS contributed to the design and the interpretation of experiments, SP planned and supervised the research and wrote the manuscript. All authors read and approved the final manuscript.

## Supplementary Material

Additional file 1 **Evidence that apoptotic PBMCs express podoplanin**. (A) Apoptotic and necrotic PBMCs express podoplanin. Podoplanin expression on viable and apoptotic PBMCs, as determined by forward and sideward scatter, was analyzed by flow cytometry (left panel, the histograms were obtained by gating on dead or live cells, as indicated by the arrows in the scatter plot). Alternatively, the cells were co-stained with podoplanin-specific antibody and the apoptosis marker annexin V or the necrosis marker 7-AAD, and staining analyzed by flow cytometry including both, live and dead cells. Black filled histogram: unstained cells, grey line: cells stained with isotype control antibody, grey filled histogram: cells stained with 18H5. (B) PBMCs were incubated with 1 μM staurosporine for the indicated times and podoplanin expression (black bars) and annexin V binding (white bars) were determined by flow cytometry. The results were confirmed in two independent experiments. Click here for file

Additional file 2 **The podoplanin-specific antibody 18H5, but not antibodies with other specificities recognize non-viable cells**. (A) CEM×174 cells were analyzed for their distribution in the forward and sideward scatter, and a gate was defined which comprised both viable and non-viable cells. (B) The CEM×174 cells were stained with the indicated monoclonal antibodies and staining of the cells gated as shown in (A) was analyzed. The results of a representative experiment are shown and were confirmed in an independent experiment. IgG1, IgG2a and IgG2b are commercially available isotype control antibodies. The anti-AU1 antibody is specific for the AU1 antigenic tag. ACE2, MER and Axl are cell surface receptors, which are used for cell entry by SARS-coronavirus (ACE2) and Ebola virus (Axl, MER). PDPN: podoplanin. Click here for file
