# Supplementary material for: Incorporation of podoplanin into HIV released from HEK-293T cells, but not PBMC, is required for efficient binding to the attachment factor CLEC-2
Source: Retrovirology. 2010 May 19;7:47. doi: 10.1186/1742-4690-7-47 (PMC2885308; doi:10.1186/1742-4690-7-47)
Supplement: Additional file 1 — Evidence that apoptotic PBMCs express podoplanin. (A) Apoptotic and necrotic PBMCs express podoplanin. Podoplanin expression on viable and apoptotic PBMCs, as determined by forward and sideward scatter, was analyzed by flow cytometry (left panel, the histograms were obtained by gating on dead or live cells, as indicated by the arrows in the scatter plot). Alternatively, the cells were co-stained with podoplanin-specific antibody and the apoptosis marker annexin V or the necrosis marker 7-AAD, and staining analyzed by flow cytometry including both, live and dead cells. Black filled histogram: unstained cells, grey line: cells stained with isotype control antibody, grey filled histogram: cells stained with 18H5. (B) PBMCs were incubated with 1 μM staurosporine for the indicated times and podoplanin expression (black bars) and annexin V binding (white bars) were determined by flow cytometry. The results were confirmed in two independent experiments. [file 1742-4690-7-47-S1.PPT]

## Slide 1
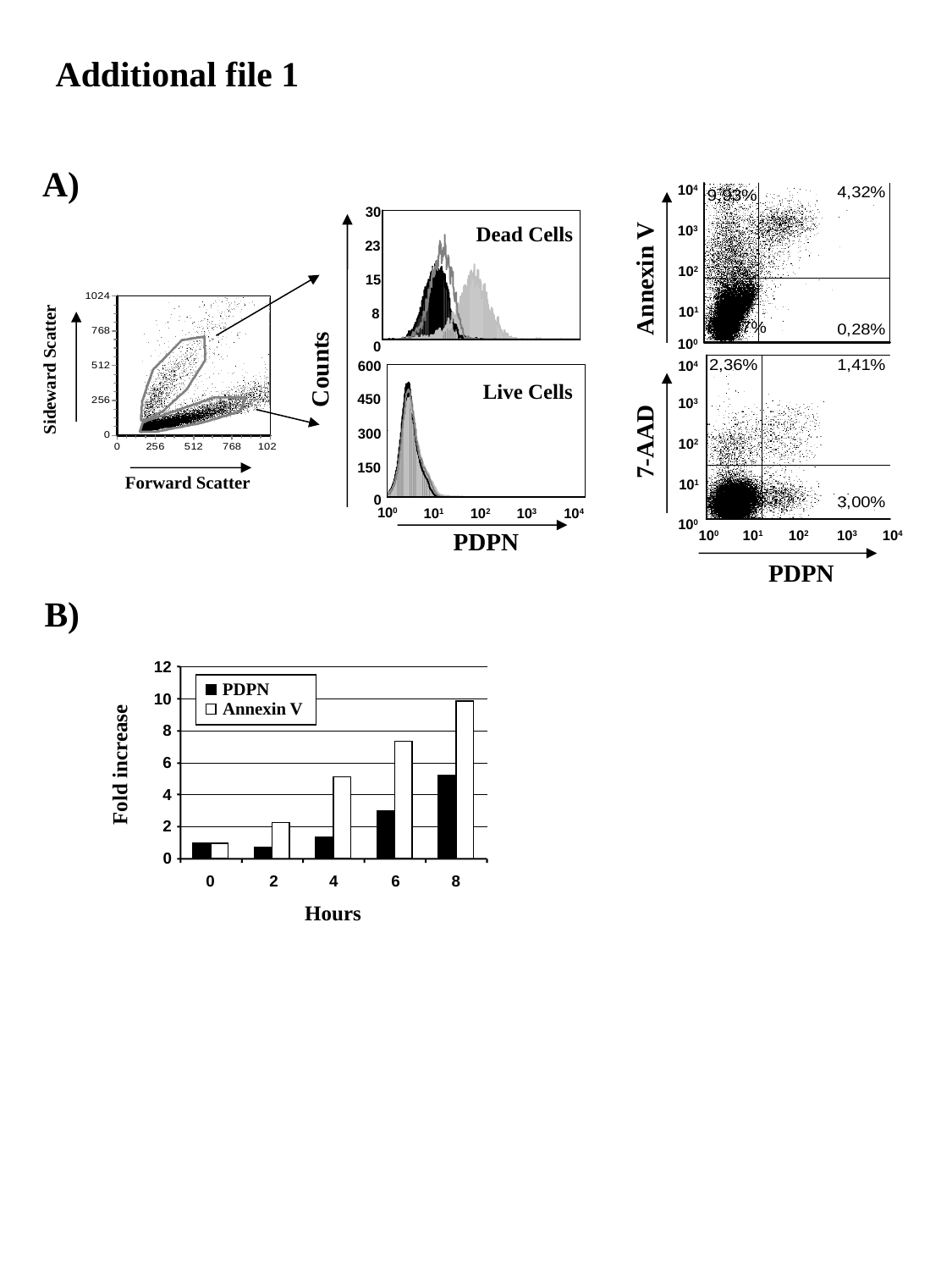

Additional file 1
A)
104
30
Dead Cells
103
23
102
Annexin V
15
101
8
100
0
Counts
104
600
Sideward Scatter
Live Cells
450
103
300
7-AAD
102
150
Forward Scatter
101
0
100
101
102
103
104
100
100
102
103
PDPN
101
104
PDPN
B)
12
PDPN
Annexin V
10
8
Fold increase
6
4
2
0
0
2
4
6
8
Hours
