# Supplementary material for: Incorporation of podoplanin into HIV released from HEK-293T cells, but not PBMC, is required for efficient binding to the attachment factor CLEC-2
Source: Retrovirology. 2010 May 19;7:47. doi: 10.1186/1742-4690-7-47 (PMC2885308; doi:10.1186/1742-4690-7-47)
Supplement: Additional file 2 — The podoplanin-specific antibody 18H5, but not antibodies with other specificities recognize non-viable cells. (A) CEM×174 cells were analyzed for their distribution in the forward and sideward scatter, and a gate was defined which comprised both viable and non-viable cells. (B) The CEM×174 cells were stained with the indicated monoclonal antibodies and staining of the cells gated as shown in (A) was analyzed. The results of a representative experiment are shown and were confirmed in an independent experiment. IgG1, IgG2a and IgG2b are commercially available isotype control antibodies. The anti-AU1 antibody is specific for the AU1 antigenic tag. ACE2, MER and Axl are cell surface receptors, which are used for cell entry by SARS-coronavirus (ACE2) and Ebola virus (Axl, MER). PDPN: podoplanin. [file 1742-4690-7-47-S2.PPT]

## Slide 1
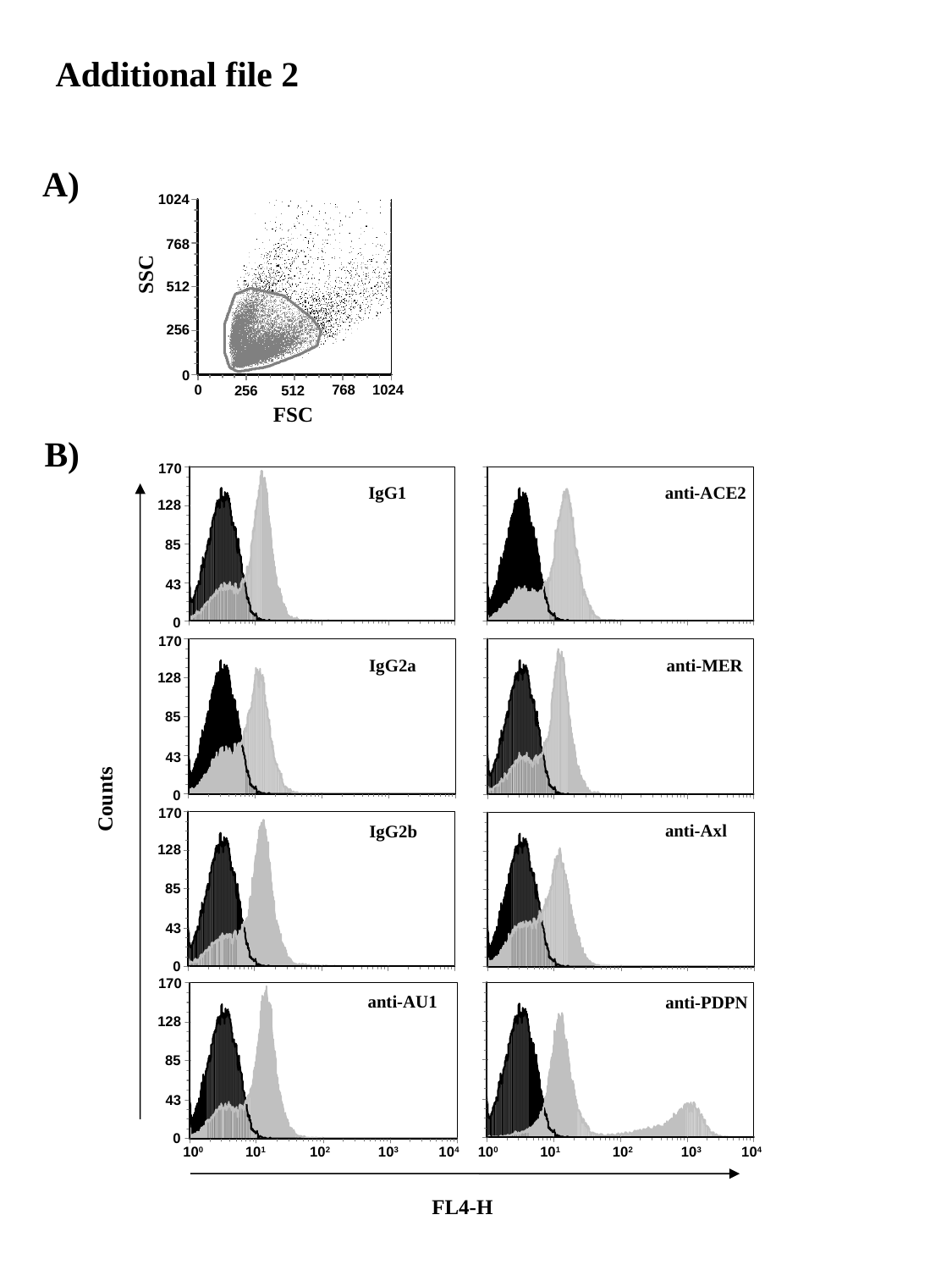

Additional file 2
A)
1024
768
SSC
512
256
0
0
768
1024
256
512
FSC
B)
170
IgG1
anti-ACE2
128
85
43
0
170
anti-MER
IgG2a
128
85
43
0
Counts
170
anti-Axl
IgG2b
128
85
43
0
170
anti-AU1
anti-PDPN
128
85
43
0
100 101 102 103 104
100 101 102 103 104
FL4-H
